# Gut microbiota and colorectal cancer: mechanistic insights, diagnostic advances, and microbiome-based therapeutic strategies

**DOI:** 10.3389/fmicb.2025.1699893

**Published:** 2025-11-20

**Authors:** Bingbing Bai, Jianing Ma, Wenlong Xu, Xiaomin Chen, Xu Chen, Chao Lv, Wei Su, Yaoxu Li, Hongyin Sun, Baoyin Zhang, Dejuan Xiang, Zhongsha Li, Yuesong Wu, Jian Sun, Mingzhu Yin

**Affiliations:** 1 Clinical Research Center (CRC), Medical Pathology Center (MPC), Cancer Early Detection and Treatment Center (CEDTC) and Translational Medicine Research Center (TMRC), Chongqing University Three Gorges Hospital, Chongqing University, Chongqing, China; 2 CQU-Ferenc Krausz Nobel Laureate Scientific Workstation, Chongqing University Three Gorges Hospital and Academy for Advanced Interdisciplinary Technology, Chongqing, China; 3 Chongqing Technical Innovation Center for Quality Evaluation and Identification of Authentic Medicinal Herbs, Chongqing, China; 4 School of Medicine, Chongqing University, Chongqing, China; 5 State Key Laboratory of Digital Medical Engineering, Key Laboratory of Biomedical Engineering of Hainan Province, School of Biomedical Engineering, Hainan University, Sanya, Hainan, China; 6 West China School of Basic Medical Sciences and Forensic Medicine, Institute of Biomedical Engineering, Sichuan University, Chengdu, China

**Keywords:** gut microbiota dysbiosis, carcinogenic mechanisms, key microbial species, multi-omics technologies, microbiota-based therapeutics

## Abstract

Colorectal cancer (CRC) is closely linked to gut microbiota dysbiosis. We synthesize evidence that carcinogenic microbes promote CRC through chronic inflammation, bacterial genotoxins, and metabolic imbalance, highlighting key pathways involving *Fusobacterium nucleatum*, *pks*^+^*Escherichia coli*, and enterotoxigenic *Bacteroides fragilis* (ETBF). Building on these mechanisms, we propose a minimal diagnostic signature that integrates multi-omics with targeted qPCR, and a pathway–therapy–microbiome matching framework to guide individualized treatment. Probiotics, fecal microbiota transplantation (FMT), and bacteriophage therapy show promise as adjunctive strategies; however, standardization, safety monitoring, and regulatory readiness remain central hurdles. We advocate a three-step path to clinical implementation—stratified diagnosis, therapy matching, and longitudinal monitoring—supported by spatial multi-omics and AI-driven analytics. This approach aims to operationalize microbiome biology into deployable tools for risk stratification, treatment selection, and surveillance, advancing toward microbiome-informed precision oncology in CRC.

## 1 Introduction

Colorectal cancer (CRC) is one of the most prevalent malignant tumors worldwide. According to the latest data released by the International Agency for Research on Cancer (IARC), the global incidence of CRC is expected to exceed 3.2 million new cases in 2040, with nearly 1.6 million deaths, ranking third among all cancers after breast and lung cancer (Morgan et al., 2022). While early detection rates are relatively high in some developed countries, such as the United States and European nations, due to well-established screening programs, the situation remains critical in developing regions including India and Africa, where screening coverage is limited and over 60% of cases are diagnosed at advanced stages (Lee and Holmes, 2023). This “high-incidence and high-mortality” pattern not only poses a significant threat to public health but also imposes a considerable burden on global healthcare systems.

With the rapid development of high-throughput sequencing, metagenomics, and metabolomics, the role of the gut microbiota in human health and disease has drawn increasing attention (Fan and Pedersen, 2020). Gut microbes maintain intestinal homeostasis and host immunity. They also contribute to CRC via chronic inflammation, bacterial genotoxins, oxidative stress, and dysregulated microbial metabolites (Dougherty and Jobin, 2023; White and Sears, 2023). Given that the colon and rectum harbor a highly dense microbial ecosystem, gut microbiota dysbiosis is now considered a pivotal environmental factor contributing to CRC onset and progression.

Throughout the multistage development of CRC, the gut microbiota interacts dynamically with the host (Kim and Lee, 2022). On one hand, specific bacterial taxa, including *Fusobacterium nucleatum* (*F. nucleatum*), *Escherichia coli* (*E. coli*), and *Bacteroides fragilis* (*B. fragilis*), are enriched in tumor tissues and can promote tumorigenesis by activating pro-inflammatory pathways, inducing DNA damage, and modulating oncogenic signaling (Wong and Yu, 2023; Ternes et al., 2020). On the other hand, strategies aimed at modulating the gut microbiome hold promise for early detection, therapeutic synergy, and even prevention of CRC (Chen and Chen, 2021).

In this review, we comprehensively summarize current advances in understanding the relationship between the gut microbiota and CRC. We focus on microbial mechanisms of tumor initiation and progression, key bacterial species with carcinogenic potential, cutting-edge microbiome detection technologies, emerging microbiota-targeted therapeutic strategies, and translational landscape, providing theoretical and translational insights for CRC prevention, early diagnosis, and precision therapy.

## 2 Mechanisms linking gut microbiota to colorectal carcinogenesis

CRC is driven by a multifactorial interplay of genetic susceptibility, environmental exposures, and gut microbiota dysbiosis. Among these factors, the gut microbiota has emerged as a pivotal environmental contributor that participates in tumor initiation, progression, and metastasis through both direct and indirect mechanisms (Cheng Y. et al., 2020). Accumulating evidence indicates that microbial imbalance disrupts host immune homeostasis and epithelial barrier integrity, while triggering chronic inflammation, genotoxic stress, oxidative damage, and metabolic dysregulation-collectively fostering a microenvironment conducive to malignant transformation (Hanus et al., 2021).

Pathogenic bacteria drive chronic inflammation that fuels precancerous growth (Zhang et al., 2023); Genotoxins damage DNA and foster driver mutations (Lai et al., 2021); Oxidative stress promotes chromosomal aberrations and perturbs key signaling pathways (Shi et al., 2021); Metabolites such as secondary bile acids, acetaldehyde, and TMAO further promote malignant transformation via immune and signaling effects (You et al., 2023).

Additionally, the gut microbiota can influence the metabolic activation or inactivation of exogenous carcinogens and chemotherapeutic agents, further modulating host tumor susceptibility. These mechanistic insights not only highlight the central role of gut microbiota dysbiosis in CRC pathogenesis but also provide a foundation for identifying microbial biomarkers and developing microbiota-targeted interventions.

In the following sections, we delineate four core mechanistic pathways underlying the contribution of gut microbes to colorectal tumorigenesis: (i) chronic inflammation driven by pathogenic bacteria; (ii) DNA damage induced by bacterial genotoxins; (iii) oxidative stress-mediated chromosomal instability; and (iv) the oncogenic influence of microbial metabolites.

### 2.1 Pathogenic bacteria and chronic inflammation

**Figure 1:**
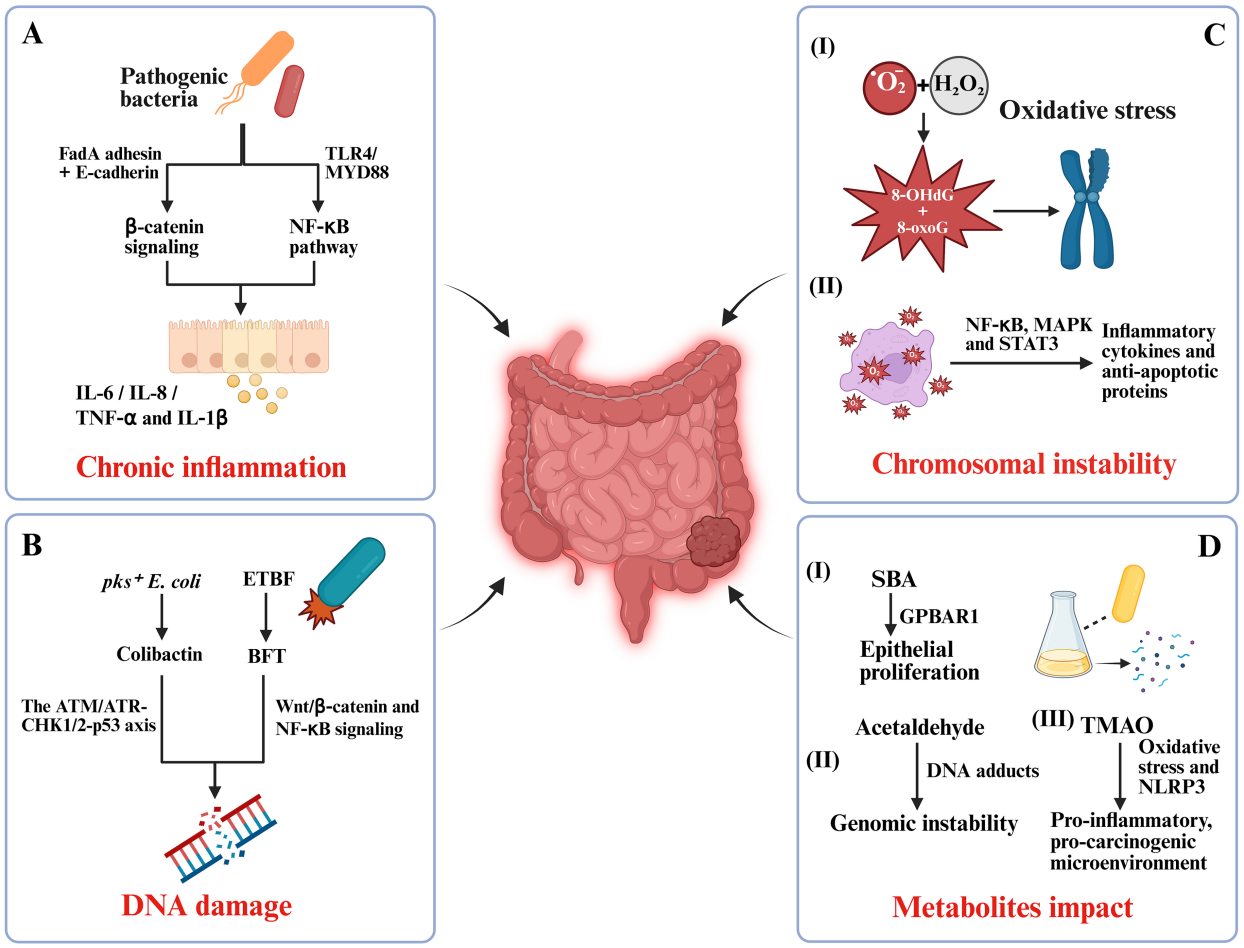
Mechanistic pathways linking gut microbiota dysbiosis to colorectal carcinogenesis. **(A)** Pathogenic bacteria-induced chronic inflammation: *Fusobacterium nucleatum* and adherent-invasive *Escherichia coli* (AIEC) trigger pro-inflammatory cascades via FadA adhesin–E-cadherin binding and TLR4/MYD88/NF-κB signaling, promoting IL-6, IL-8, TNF-α, and IL-1β secretion. **(B)** Bacterial genotoxins and DNA damage: *pks^+^ E. coli* produces colibactin that induces double-strand breaks through the ATM/ATR-CHK1/2-p53 axis, while enterotoxigenic *Bacteroides fragilis* (ETBF) secretes BFT toxin, activating Wnt/β-catenin and NF-κB pathways. **(C)** Oxidative stress and chromosomal instability: Reactive oxygen species (ROS) from *Enterococcus faecalis* cause oxidative DNA lesions (e.g., 8-OHdG, 8-oxoG) and activate NF-κB, MAPK, and STAT3, enhancing inflammatory and anti-apoptotic signaling. **(D)** Microbial metabolite effects: Secondary bile acids (SBA), acetaldehyde, and trimethylamine N-oxide (TMAO) drive epithelial proliferation, genomic instability, oxidative stress, and a pro-carcinogenic microenvironment.

Chronic inflammation is one of the most prominent pro-tumorigenic factors within the colorectal tumor microenvironment. Various pathogenic gut bacteria initiate and sustain mucosal inflammatory responses through multiple mechanisms, including epithelial adhesion, toxin secretion, and immune activation (Lee et al., 2022; Jergens et al., 2021). Among them, *F. nucleatum* is the most extensively studied pro-inflammatory bacterium in CRC. Its FadA adhesin binds to E-cadherin on host intestinal epithelial cells, triggering β-catenin signaling and inducing the expression of inflammatory cytokines such as IL-6 and IL-8, thereby establishing a localized inflammatory niche (Li et al., 2022). Beyond β-catenin pathway engagement via FadA–E-cadherin, downstream nuclear β-catenin activity upregulates prototypical targets including MYC and CCND1 (Cyclin D1), which promote proliferation and cell-cycle progression. In parallel, TLR4/MYD88/NF-κB signaling augments pro-inflammatory cytokines and cooperates with Wnt pathway, together reinforcing epithelial proliferation and a tumor-permissive niche (Figure 1A) (Hu et al., 2021).

Certain *E. coli* strains, including adherent-invasive *E. coli* (AIEC), are also frequently enriched in patients with inflammatory bowel disease and CRC. These bacteria can invade epithelial cells, evade immune clearance, and continuously stimulate the activation of T cells and dendritic cells, leading to the release of pro-inflammatory mediators such as TNF-α and IL-1β (Figure 1A) (Viladomiu et al., 2021). This persistent, low-grade inflammation compromises epithelial barrier integrity, promotes abnormal epithelial proliferation, and increases the likelihood of mutational accumulation.

Sustained chronic inflammation further promotes tumorigenesis by reshaping the immune microenvironment. It recruits myeloid-derived suppressor cells (MDSCs) and polarizes tumor-associated macrophages (TAMs), thereby facilitating immune evasion (Siddiqui and Glauben, 2022; Li et al., 2020). Moreover, inflammatory cytokines upregulate cyclooxygenase-2 (COX-2) expression and enhance prostaglandin E2 (PGE_2_) production, which collectively stimulate angiogenesis and extracellular matrix degradation (Finetti et al., 2020). These processes generate a supportive “fertile soil” for tumor invasion and distant metastasis.

### 2.2 Genotoxins and DNA damage

Certain gut bacterial strains contribute to colorectal carcinogenesis by producing genotoxic compounds that directly compromise genomic integrity, representing a key early event in tumor initiation (Cao et al., 2022). A prime example is *E. coli* strains harboring the *pks* pathogenicity island, which encodes colibactin. Colibactin is an alkylating genotoxin that binds the DNA minor groove. It induces double-strand breaks and activates DDR pathways, including the ATM/ATR–CHK1/2–p53 axis (Figure 1B) (Gerstberger et al., 2025). In murine models, colonization with *pks^+^ E. coli* significantly increases γ-H2AX foci formation and the incidence of microadenomas in the intestinal epithelium, confirming its carcinogenic potential (Iftekhar et al., 2021).

Enterotoxigenic *B. fragilis* (ETBF) secretes BFT, a zinc-dependent metalloprotease that cleaves epithelial E-cadherin. This proteolysis disrupts the cadherin–catenin complex, releasing β-catenin from the adherens junction and permitting its nuclear translocation, where it partners with TCF/LEF to drive transcription of canonical Wnt targets (MYC, CCND1/Cyclin D1) (Lee et al., 2022). Thus, the pathway is not a direct ligand-like “activation” of Wnt; rather, E-cadherin cleavage is the proximal event that enables β-catenin–dependent transcription, alongside BFT-associated NF-κB signaling and barrier disruption (Figure 1B) (Curti and Campaner, 2021).

Importantly, these genotoxins often act synergistically with chronic inflammation, creating a mutagenic microenvironment. By promoting oxidative stress, epigenetic alterations, and chromosomal instability, they drive epithelial cells into a “high-variability” state that greatly increases the likelihood of malignant transformation (Wang and Fu, 2023).

### 2.3 Oxidative stress and chromosomal abnormalities

Oxidative stress, resulting from the excessive accumulation of reactive oxygen species (ROS) and reactive nitrogen species (RNS), is a critical molecular driver in colorectal carcinogenesis. Gut microbiota dysbiosis can markedly alter the redox balance of the intestinal microenvironment, promoting DNA damage and genomic instability (Hamamah et al., 2024). In host–microbe interactions, *Enterococcus faecalis* (*E. faecalis*) generates ROS, primarily superoxide anions (O₂^−^) and hydrogen peroxide (H₂O₂), which induce oxidative DNA damage in the host. This damage is characterized by the formation of 8-hydroxy-2′-deoxyguanosine (8-OHdG), along with oxidized bases (e.g., 8-oxoguanine, 8-oxoG) and strand breaks that challenge DNA repair pathways. Under mismatch repair (MMR) deficiency, the processing of oxidative mismatches—such as 8-oxoG: A mispairs—becomes error-prone and inadequately corrected, thereby increasing mutational burden and promoting microsatellite instability (MSI). Thus, crosstalk between ROS and MMR mechanistically links *E. faecalis*–driven oxidative stress to genomic instability and may account for the heightened susceptibility of dMMR/MSI-high contexts to microbe-induced mutagenesis (Figure 1C) (Tang et al., 2022; Fang et al., 2024). Such oxidative base modifications and strand breaks, particularly when they occur in tumor suppressor genes (e.g., *TP53*, *APC*) or proto-oncogene regions, can lead to point mutations, misrepaired double-strand breaks, and structural chromosomal abnormalities such as translocations, deletions, or amplifications (Kavec et al., 2022). These alterations are detectable even at early adenoma stages, highlighting oxidative stress as a pivotal tumor-initiating factor.

Beyond direct genotoxicity, oxidative stress activates multiple pro-tumorigenic signaling pathways, including NF-κB, MAPK, and STAT3, which upregulate inflammatory cytokines and anti-apoptotic proteins, thereby facilitating the survival of genetically damaged cells (Figure 1C) (Alanazi et al., 2024; Mukherjee et al., 2022). A persistent positive feedback loop between oxidative stress and inflammation forms a “pro-carcinogenic ecosystem,” particularly evident in colitis-associated colorectal cancer (CAC) (Bardelčíková et al., 2023).

Collectively, microbially induced oxidative stress not only drives DNA damage and chromosomal instability but also shapes an inflammatory and anti-apoptotic tumor microenvironment that favors malignant progression (Neganova et al., 2021).

### 2.4 Microbial metabolites and their impact

Microbial metabolites serve as critical mediators of host–microbe interactions and exert profound effects on intestinal epithelial homeostasis, immune regulation, and tumorigenesis. Among these, secondary bile acids (SBAs), acetaldehyde, and TMAO are strongly implicated in CRC development (Figure 1D) (Zhang et al., 2021).

SBAs, such as deoxycholic acid (DCA) and lithocholic acid (LCA), are generated through the microbial transformation of primary bile acids, primarily by *Clostridium* species. Elevated levels promote epithelial proliferation, inhibit apoptosis, and enhance adhesion and migration via GPBAR1/TGR5 (Qi Y. et al., 2022). High concentrations of DCA can also trigger endoplasmic reticulum (ER) stress and activate the unfolded protein response (UPR), shifting the balance toward cell survival and facilitating malignant transformation (Oakes, 2020).

Acetaldehyde, a highly reactive and mutagenic metabolite generated by microbial alcohol dehydrogenase during ethanol metabolism, directly forms DNA adducts, induces interstrand crosslinks, and interferes with base excision repair, cumulatively driving mutational burden and genomic instability (Figure 1D) (Oakes, 2020).

TMAO, a gut microbiota-derived metabolite of choline, L-carnitine, and phosphatidylcholine, has emerged as a systemic tumor-promoting factor. It can disrupt cellular energy metabolism, induce oxidative stress, and activate the NLRP3 inflammasome, thereby facilitating a pro-inflammatory, pro-carcinogenic microenvironment (Figure 1D) (Lei et al., 2023).

Additionally, β-glucuronidase (GUS) activity is markedly increased in the fecal samples of CRC patients. This enzyme hydrolyzes glucuronide conjugates of carcinogens excreted in bile, reactivating these compounds within the intestinal lumen and enhancing DNA damage (Hillege et al., 2024). Collectively, these microbial metabolites function not only as mechanistic effectors of tumor initiation and progression but also as potential biomarkers and therapeutic targets for metabolic interventions in CRC.

## 3 Key microbial species and their CRC-specific mechanisms

Comparative analyses show a marked microbial imbalance in CRC. Pro-carcinogenic species are enriched, while commensal or beneficial bacteria are depleted. Pathogenic taxa such as *F. nucleatum*, toxigenic *E. coli*, and enterotoxigenic *B. fragilis* are significantly enriched in CRC patients, whereas beneficial microbes including *Bifidobacterium* and *Lactobacillus* species are markedly reduced (Senthakumaran et al., 2023; Qu et al., 2023). This gut microbiota dysbiosis not only disrupts intestinal homeostasis but also impairs epithelial barrier integrity and weakens immune surveillance, creating a permissive niche for microbial colonization and tumor initiation.

High-resolution analyses of tumor-associated microbiota demonstrate that some pathogens exhibit striking tissue tropism. For example, *F. nucleatum* can accumulate within adenomas and carcinoma tissues at levels several-fold to hundreds of times higher than in adjacent normal mucosa (Wang and Fang, 2022). Concomitantly, CRC patients typically show reduced microbial diversity, which diminishes ecological resilience and heightens susceptibility to environmental perturbations such as high-fat diets, antibiotic exposure, or chemotherapy (Yang et al., 2020; Kenneth et al., 2025). This dysbiotic state favors the expansion of pathobionts and triggers pro-carcinogenic processes through chronic inflammation, genotoxic insult, and metabolite-driven signaling (Rossi et al., 2020; Finetti et al., 2020).

In the following sections, we highlight six key microbial species or genera with well-characterized contributions to CRC pathogenesis-*F. nucleatum*, *E. coli*, *B. fragilis*, *E. faecalis*, *Streptococcus bovis* (*S. bovis*), and *Peptostreptococcus anaerobius* (*P. anaerobius*). We summarize their molecular mechanisms, associated host signaling pathways, and clinical implications, providing a foundation for the development of microbiota-based biomarkers and targeted interventions.

### 3.1 Fusobacterium nucleatum

*F. nucleatum* is an anaerobic Gram-negative bacterium that is commonly found in the oral cavity but is consistently enriched in the colorectal tumors of patients with CRC. Its abundance is particularly elevated in adenomas and carcinoma tissues compared with adjacent normal mucosa, suggesting an active role in early tumorigenesis and progression. Mechanistically, *F. nucleatum* contributes to CRC through three interconnected processes: inflammation activation, signaling pathway modulation, and immune evasion (Wang and Fang, 2022).

First, *F. nucleatum* promotes a pro-inflammatory tumor microenvironment. Its surface lipopolysaccharide (LPS) interacts with Toll-like receptor 4 (TLR4) on colonic epithelial cells, activating the TLR4/MYD88/NF-κB signaling axis (Luo et al., 2025). This leads to the transcription of pro-inflammatory cytokines and upregulation of oncogenic microRNA-21 (miR-21) (Sun et al., 2021). miR-21 downregulates RASA1, releasing suppression of the RAS-MAPK pathway, thereby enhancing tumor cell proliferation, invasion, and resistance to apoptosis (Bhere et al., 2020).

Second, the bacterial adhesin FadA binds to E-cadherin on epithelial cells, triggering β-catenin nuclear translocation and activation of the Wnt/β-catenin pathway (Jiang et al., 2024). This signaling cascade accelerates cell cycle progression and abnormal epithelial proliferation, key events in the adenoma-to-carcinoma sequence. Additionally, *F. nucleatum* modulates host lipid metabolism by promoting the production of linoleic acid-derived 12,13-epoxyoctadecenoic acid (12,13-EpOME), which facilitates epithelial-mesenchymal transition (EMT) and enhances metastatic potential (Kong et al., 2021).

Third, *F. nucleatum* exerts dual effects on the immune microenvironment. While it can activate the STING pathway to enhance dendritic cell antigen presentation, it simultaneously suppresses natural killer (NK) cell cytotoxicity and elevates ROS levels, thereby creating a tumor-permissive niche (Gao et al., 2021). This immunomodulatory balance underlies its capacity to promote tumor progression and immune evasion.

Collectively, *F. nucleatum* acts as a multifaceted driver of CRC through the induction of chronic inflammation, activation of oncogenic signaling pathways, and remodeling of the immune microenvironment. Its tissue enrichment and mechanistic links to tumorigenesis position it as a promising biomarker for early CRC detection and as a potential target for microbiota-based therapeutic interventions.

### 3.2 Escherichia coli

*E. coli* is a facultative anaerobic Gram-negative bacterium that exists as both a commensal and an opportunistic pathogen in the human gut. Certain pathogenic strains, particularly those harboring the *pks* genomic island, have been strongly implicated in colorectal carcinogenesis. The *pks* island encodes colibactin, a genotoxic secondary metabolite capable of alkylating DNA and inducing interstrand crosslinks and double-strand breaks (Pleguezuelos-Manzano et al., 2020; Gerstberger et al., 2025). These lesions activate canonical DDR pathways, including the ATM/ATR-CHK1/2-p53 axis, and lead to mutational accumulation and genomic instability if repair is incomplete (Pleguezuelos-Manzano et al., 2020; Huber et al., 2024). Animal studies have demonstrated that colonization with *pks*^+^ *E. coli* elevates γ-H2AX levels and promotes early adenoma formation, directly linking colibactin activity to tumor initiation.

Beyond its direct genotoxicity, *E. coli* contributes to CRC through inflammatory and signaling mechanisms. The bacterium can activate Wnt/β-catenin and STAT3 pathways, driving epithelial proliferation and anti-apoptotic signaling (Trejo-Solís et al., 2021; Zhou et al., 2021). Other virulence factors, such as Shiga toxins and hemolysins, exacerbate epithelial barrier injury, enhance ROS production, and amplify chronic inflammation (Warr et al., 2020). Interaction with host immune cells further accelerates tumor-promoting inflammation: *E. coli* engages the TLR4/NF-κB signaling axis to induce pro-inflammatory cytokines, including IL-6, IL-8, and TNF-α, which sustain tumor-promoting microenvironments and facilitate angiogenesis (Lan et al., 2022; Zhuang et al., 2020).

High abundance of these strains has been associated with worse overall survival and higher recurrence rates, highlighting their potential as prognostic biomarkers. Given their multifaceted roles in DNA damage, signaling activation, and immune modulation, pathogenic *E. coli* strains represent not only key drivers of CRC but also compelling targets for microbiome-focused diagnostic and therapeutic strategies.

### 3.3 Bacteroides fragilis

*B. fragilis* is a common anaerobic bacterium in the human gut, but its pathogenic potential is largely associated with toxigenic strains, collectively known as ETBF. ETBF secretes BFT, a zinc-dependent metalloprotease that cleaves E-cadherin at the epithelial junctions, compromising barrier integrity and facilitating inflammatory infiltration (Lukiw, 2020). This early disruption of epithelial homeostasis is a critical initiating event in microbe-driven tumorigenesis.

Mechanistically, BFT drives CRC progression through both inflammatory and oncogenic signaling pathways. First, BFT induces COX-2 expression and promotes PGE_2_ production, fostering a state of chronic inflammation that supports epithelial proliferation and survival (Wei et al., 2022). Second, BFT activates the STAT3 signaling cascade, enhancing anti-apoptotic gene expression and promoting the maintenance of cancer stem-like properties (Yang J. et al., 2024). ETBF colonization also reshapes local immune responses: it suppresses IL-2 expression in regulatory T cells, promotes the differentiation of Th17 cells, and elevates IL-17 levels, which in turn induces IL-6 production (Jo et al., 2023). This creates a self-sustaining IL-6/STAT3 positive feedback loop that amplifies tumor-promoting inflammation (Pandey et al., 2024).

Evidence from animal models further supports the carcinogenic potential of ETBF. Colonization with ETBF accelerates colonic epithelial proliferation, induces sustained mucosal inflammation, and promotes adenoma formation (Yang J. et al., 2024). Given its well-defined role at the intersection of inflammation and oncogenesis, ETBF is increasingly recognized as both a potential microbial biomarker for CRC risk and a candidate target for microbiome-focused preventive or therapeutic interventions (Zamani et al., 2020).

### 3.4 Enterococcus faecalis

*E. faecalis* is a Gram-positive, facultative anaerobe that commonly inhabits the human gut and oral cavity (Madani et al., 2024). Although long regarded as a commensal—and in some contexts explored as a probiotic candidate—accumulating evidence indicates a strain-dependent, dual role in CRC (Elnar and Kim, 2025; Daca and Jarzembowski, 2024). Beneficial or food-derived strains can support barrier function and immune homeostasis, whereas pathogenic or clinical isolates harbor virulence and antimicrobial-resistance determinants and are capable of driving pro-carcinogenic biology (Zhang L. et al., 2024).

Mechanistically, *E. faecalis* contributes to tumor promotion through intertwined inflammatory, oxidative, and genotoxic processes. Certain isolates generate high levels of reactive oxygen species (ROS; superoxide and hydrogen peroxide), producing oxidized DNA bases (e.g., 8-oxoG/8-OHdG) and strand breaks that challenge canonical repair pathways (Kouhzad et al., 2025). This oxidative stress operates alongside mucosal inflammation to create a microenvironment favorable to malignant transformation and progression (Catalano et al., 2025). Importantly, MMR status modulates the genomic consequences of *E. faecalis*–derived ROS: in MMR-deficient settings, error-prone processing of oxidative mismatches (such as 8-oxoG: A mispairs) is inadequately corrected, amplifying mutational burden and microsatellite instability, and thereby linking microbial ROS directly to genomic instability in susceptible hosts (Sun et al., 2025).

Beyond oxidative injury, *E. faecalis* can influence epithelial signaling and cell-cycle control. ROS-driven kinase activation and inflammatory transcriptional programs converge on pathways that enhance proliferation and survival, and may cooperate with other microbe-host axes present in CRC lesions (Hong et al., 2024). The net effect is a genotoxic, pro-inflammatory niche that lowers the threshold for oncogenic evolution and can attenuate responses to cytotoxic therapy (Catalano et al., 2025).

These features have practical implications. First, because oncogenic potential varies by strain, future translational work should incorporate genomic and virulence profiling (including toxin gene content and antimicrobial-resistance genes) when *E. faecalis* is considered in diagnostics or as a therapeutic adjunct. Second, in mechanistic and clinical studies, MMR context should be specified *a priori*, as dMMR/MSI-high tumors may be particularly vulnerable to ROS-mediated mutagenesis (Sun et al., 2025). Third, where *E. faecalis* is implicated in gut microbiota dysbiosis, narrow-spectrum or sequencing-guided antimicrobial strategies, coupled with restoration of barrier function and short-chain fatty acid production, may mitigate collateral damage while addressing the offending strains. Finally, targeted qPCR panels and metagenomic readouts that resolve *E. faecalis* at the strain level can support peri-operative monitoring and help relate microbial dynamics to treatment tolerance and outcome.

In sum, *E. faecalis* exemplifies how a common commensal can become a context- and strain-specific contributor to CRC pathogenesis. Recognizing its dual identity—and explicitly accounting for strain heterogeneity and host MMR status—will be essential for accurate risk attribution and for the rational design of microbiome-informed prevention and therapeutic strategies.

### 3.5 Streptococcus bovis

*Streptococcus bovis*/*Streptococcus equinus* complex (SBSEC) remains strongly associated with colorectal neoplasia, but recent evidence favors risk estimates from meta-analyses over broad prevalence ranges. A 2023 systematic review reported that patients with SBSEC bacteremia were 3.73-fold more likely to harbor underlying CRC than those without bacteremia (RR 3.73, 95% CI 2.79–5.01) (Ouranos et al., 2023). In pooled case–control data, CRC cases were ~2.27-fold more likely to demonstrate SBSEC colonization or anti–*S. gallolyticus* IgG responses than controls. The association appears genospecies-dependent, being stronger for *S. gallolyticus* subsp. *gallolyticus* than for other SBSEC members. These data support the clinical recommendation that patients with SBSEC bacteremia or infective endocarditis undergo routine colonoscopic evaluation to detect CRC and guide early management (Ouranos et al., 2023).

The tumor-promoting mechanisms of *S. bovis* are multifaceted, involving both inflammatory and proliferative pathways (Karpiński et al., 2022). The bacterium can stimulate the NF-κB pathway, inducing the secretion of pro-inflammatory cytokines such as IL-6, IL-8, and TNF-α, thereby fostering a chronic inflammatory microenvironment that facilitates tumor initiation and progression (Neumann et al., 2021). In parallel, *S. bovis* can engage TLR2 and TLR4 signaling, driving the recruitment of CD11b^+^ TLR4^+^ monocytes to the tumor site, which contributes to local immune suppression and facilitates immune evasion by cancer cells (Huang et al., 2020).

Beyond immune modulation, *S. bovis* can directly influence epithelial cell proliferation by activates the Ras–Raf–MEK–ERK and JNK/p38 MAPK pathways, accelerating cell-cycle progression and enhancing oncogenic signaling (Xiao et al., 2020). This dual action-chronic inflammation coupled with aberrant epithelial proliferation-creates a pro-tumorigenic niche.

Notably, *S. bovis* has also been detected in the bloodstream of CRC patients, suggesting a potential role in tumor-associated bacteremia. Enhanced bacterial adhesion to endothelial surfaces may promote vascular permeability, support tumor-associated angiogenesis, and facilitate distant metastasis (Laupland et al., 2023). Owing to its early enrichment during CRC development and its detectability via blood cultures or serological testing, *S. bovis* has been proposed as a candidate biomarker for high-risk CRC screening (Thind et al., 2021).

Translationally, these data support a genospecies-aware, oncology-aligned approach. SBSEC bacteremia—or infective endocarditis—should trigger colonoscopic evaluation to detect occult CRC and enable early management, consistent with recent meta-analytic risk estimates (Ouranos et al., 2023). Diagnostic workups should aim for lineage-level resolution (e.g., *S. gallolyticus* subsp. *gallolyticus*) and, where feasible, integrate virulence profiling into future qPCR panels for risk stratification. Within oncology pathways, SBSEC positivity should prompt assessment of metastatic risk and source control (mucosal lesions, dental foci, biliary tract) alongside guideline-concordant antimicrobials, reflecting modern evidence on genospecies-specific risk.

Looking ahead, embedding SBSEC diagnostics and management within oncology workflows enables earlier case finding, molecularly informed risk tiers, and longitudinal surveillance that links infection control to CRC prevention, staging, and treatment planning. Priorities for future research include high-resolution characterization of virulence factors, host–receptor interactions, and strain-specific pathogenicity, together with the co-development of deployable qPCR-based SBSEC panels that can be integrated into perioperative and adjuvant care pathways.

### 3.6 Peptostreptococcus anaerobius

*P. anaerobius* is a Gram-positive anaerobic coccus that commonly resides in the human oral cavity and gastrointestinal tract. In recent years, this bacterium has gained attention for its potential role in CRC progression. Multiple independent metagenomic and tissue-based studies have demonstrated its significant enrichment in CRC patients, particularly within tumor-adjacent mucosa and intratumoral regions, suggesting a spatially specific association with tumorigenesis (Conde-Pérez et al., 2024).

Mechanistically, *P. anaerobius* promotes CRC through several interrelated pathways. It activates TLR2 and TLR4 mediated signaling, triggering the NF-κB pathway and upregulating pro-inflammatory cytokines such as IL-1β, TNF-α, and IL-6, thereby fostering a chronic inflammatory microenvironment conducive to malignant transformation (Shen et al., 2024). Concurrently, the bacterium interacts with membrane cholesterol to form cholesterol-rich microdomains that facilitate the activation of Ras–Raf–MEK–ERK and JNK/p38 MAPK signaling cascades (Bahar et al., 2023). These pathways enhance epithelial cell proliferation, migration, and survival, supporting tumor progression.

In addition to its pro-inflammatory and pro-proliferative effects, *P. anaerobius* can exacerbate genomic instability by increasing ROS production and downregulating DNA repair enzymes, resulting in oxidative DNA damage and accelerated cell-cycle progression via upregulation of Cyclin D1 (Gupta et al., 2023). This genotoxic and proliferative environment establishes a fertile ground for carcinogenesis. Furthermore, emerging evidence suggests that *P. anaerobius* may impair antitumor immunity by reducing CD8^+^ T-cell infiltration and inducing PD-L1 expression on tumor cells, thereby facilitating immune evasion (Wang et al., 2020).

These mechanisms have direct therapeutic relevance. Associations between *P. anaerobius* abundance and attenuated responses to immune checkpoint blockade or cytotoxic chemotherapy point to a microbiome-linked axis of resistance mediated by inflammatory remodeling of the tumor microenvironment and the expansion of myeloid-derived suppressor cells. Where resources allow, baseline microbial profiling should be incorporated into treatment planning—particularly for immunotherapy or irinotecan-based regimens—to anticipate resistance trajectories and to guide supportive measures.

The organism’s dependency on cholesterol-enriched membrane domains also highlights tractable points of intervention. Approaches under investigation include membrane-lipid remodeling and small-molecule blockade of microdomain formation to disrupt adhesion, signaling, and biofilm stability, with the aim of restoring sensitivity to systemic therapy. In clinical practice, when a high *P. anaerobius* signal is detected by tissue or fecal qPCR/metagenomics, it is reasonable to prioritize regimens that pair anticancer therapy with anti-inflammatory measures and targeted antimicrobials. Sequencing-guided, narrow-spectrum strategies may limit gut microbiota dysbiosis; longitudinal qPCR can then be used to track organismal clearance and relate microbial dynamics to immune activation and clinical response.

From a prognostic perspective, *P. anaerobius* clusters within chemoresistance-associated microbiome profiles, suggesting value as a predictive biomarker for treatment response and outcome (Xing et al., 2025). Future work should define strain-level virulence factors and host–pathogen interactions, test mechanism-anchored adjuncts in biomarker-selected cohorts, and embed microbial–immune monitoring into peri-operative and adjuvant pathways to establish causal links between microbial modulation and restoration of antitumor immunity. This integrated view positions *P. anaerobius* alongside *F. nucleatum* and SBSEC as a microbiome target with clear diagnostic and therapeutic implications.

## 4 Gut microbiome detection technologies

Advances in molecular biology and multi-omics have dramatically expanded our ability to characterize the gut microbiome, enabling both taxonomic and functional profiling of microbial communities (Deissova et al., 2023). These methods have not only deepened our understanding of microbial contributions to CRC but also opened avenues for early detection, therapeutic monitoring, and precision interventions. Contemporary microbiome detection strategies can be broadly categorized into nucleic acid-based sequencing, functional and metabolic analyses, and imaging-based spatial profiling (Table 1).

**Table 1:** Summary of gut microbiome detection technologies and their clinical relevance in CRC.

Technology	Principle and key features	Advantages	Limitations	Clinical relevance in CRC	Approximate cost	Clinical accessibility
16S rRNA sequencing	Amplification of conserved and variable regions of 16S rRNA for bacterial taxonomic profiling (Kameoka et al., 2021)	Cost-effective; High throughput; Suitable for large cohorts (Durazzi et al., 2021)	Limited resolution (genus/species); No functional data; Cannot detect viruses/fungi	Initial microbiome profiling; Dysbiosis and biomarker discovery	Low	Routinely outsourced at tertiary centers
Shotgun metagenomics	Untargeted sequencing of all microbial DNA to identify species/strains and functional genes	High resolution; Detects functional genes, AMR, virulence	High cost; Data-intensive; Requires complex bioinformatics	Strain-level CRC biomarker discovery; Detection of virulence genes (*pks*, *bft*) (Yan et al., 2022)	Medium-High	Predominantly research settings
Metatranscriptomis	Sequencing of microbial mRNA to capture actively expressed functions	Provides “real-time” activity; Detects functional shifts	RNA instability; High technical demand; Expensive	Identifies metabolically active pathogens; Monitors therapy response	High	Predominantly research settings
Metabolomics	Profiling of microbe-derived metabolites in stool, blood, or urine using MS or NMR (Chen et al., 2021)	Reflects functional output; Links microbiome to host metabolism	Cannot pinpoint microbial source; Requires multi-omics integration	Detects SCFA loss, bile acid/TMAO elevation; Biomarker for CRC risk	Medium	Predominantly research settings
FISH & RNAscope-FISH	Fluorescent probes hybridized to microbial nucleic acids within tissue sections	Spatial resolution; Preserves host-microbe context	Endpoint analysis; Requires tissue optimization	Visualizes tumor-associated microbes; Links microbiota to histopathology (Castellarin et al., 2012; Strauss et al., 2011)	Medium	Research-pathology collaboration
qPCR	Targeted detection of specific microbes or functional genes using fluorescent probes	Highly sensitive; Fast; Low cost; Easy clinical adoption	Only known targets; Limited multiplexing	Pre/postoperative monitoring; Rapid detection of *F. nucleatum*, *pks*, *bft* (Yao et al., 2021)	Low	Clinical routine
Advanced imaging (IHC, EM, c-FIB/SEM)	Combines ultrastructural and fluorescent imaging for 3D host-microbe mapping (Weiner and Enninga, 2019)	High-resolution spatial biology; Detects intracellular microbes	Technically complex; Low throughput; Primarily research use	Reveals bacterial niches and microenvironment interactions	–	–
Spatial transcriptomics	Integrates bacterial RNA detection with host gene/protein mapping in situ	Maps microbes to immune/tumor niches; Multi-omics integration	High cost; Requires specialized platforms	Identifies immunosuppressive margins; Guides microbiome-informed therapy (Galeano Niño et al., 2022)	Very high	Predominantly research settings

### 4.1 16S rRNA and metagenomic sequencing

16S rRNA gene sequencing remains the most widely used approach for initial gut microbiota profiling. By amplifying conserved and variable regions of the 16S rRNA gene, this method provides insights into microbial community structure, diversity, and composition (Kameoka et al., 2021). It is cost-effective and suitable for large-scale population studies or preliminary CRC microbiome screening (Durazzi et al., 2021). Several studies have demonstrated enrichment of *F. nucleatum* and other pathobionts, alongside depletion of *Bifidobacterium* and *Lactobacillus*, in CRC patients (Yuan et al., 2022; Qi Z. et al., 2022). However, 16S sequencing is limited in taxonomic resolution (typically to genus or species level), cannot detect viruses or fungi, and does not provide direct functional information (Matchado et al., 2023).

Shotgun metagenomic sequencing overcomes these limitations by performing untargeted, whole-genome sequencing of all microbial DNA in a sample. This approach enables species and strain-level identification and captures functional gene content, including virulence factors, antibiotic resistance genes, and key metabolic pathways (Durazzi et al., 2021; Xie et al., 2023). In CRC research, metagenomics has been instrumental in linking specific microbial genes-such as the *pks* island encoding colibactin and *bft* encoding *B. fragilis* toxin-to tumorigenesis (Yan et al., 2022). Despite its strengths, metagenomics is data-intensive, costly, and requires rigorous sample processing and bioinformatics pipelines.

### 4.2 Metatranscriptomics and metabolomics

Beyond static DNA-based analyses, metatranscriptomics captures the active transcriptional state of the microbiome, providing a real-time snapshot of microbial activity. This approach reveals which genes are actively expressed in specific clinical contexts, such as chemotherapy exposure or post-surgical recovery, and can identify functionally active pathogens or beneficial commensals (Uehara et al., 2022). For instance, increased GUS expression during irinotecan treatment has been linked to drug toxicity, while *Bifidobacterium longum* activity correlates with anti-inflammatory effects (Chung et al., 2020).

Metabolomics, using mass spectrometry or nuclear magnetic resonance, interrogates the small-molecule metabolites produced by the gut microbiome (Chen et al., 2021). This provides critical insights into host-microbe metabolic crosstalk, including alterations in short-chain fatty acids (SCFAs), secondary bile acids, and TMAO associated with CRC progression (Coker et al., 2022). Integrating metagenomics and metabolomics enables the construction of microbiome-metabolite-disease networks, offering a robust framework for biomarker discovery and therapeutic monitoring (Gao et al., 2022).

### 4.3 Quantitative PCR

Quantitative PCR (qPCR) serves as a targeted, rapid, and highly sensitive technique for detecting known microbial species or virulence genes. In CRC-related applications, qPCR is commonly employed to detect *F. nucleatum*, *toxigenic E. coli*, and *B. fragilis*, as well as *pks* and *bft* genes (Yao et al., 2021). This method is well-suited for dynamic monitoring using fecal or tissue samples, supporting preoperative screening, postoperative surveillance, and evaluation of microbiota-targeted interventions. Although qPCR is limited to known targets, its low cost, high sensitivity, and rapid turnaround make it a valuable complement to high-throughput sequencing, especially in clinical workflows (Senthakumaran et al., 2024).

Despite its utility, qPCR is susceptible to false-positive results arising from (i) sample contamination or carryover amplicons; (ii) non-specific amplification/primer–dimer artifacts; (iii) homology-driven cross-reactivity with related taxa; (iv) detection of DNA from non-viable cells or extracellular DNA (especially in low-biomass matrices); and (v) thresholding bias (liberal Ct cutoffs) and multiple-target testing without correction. To reduce these risks in CRC workflows, we recommend strict pre-analytical controls tailored for low-biomass work (e.g., negative extraction/NTCs, unidirectional workflow) together with transparent contamination reporting, as emphasized in recent guidance for low-biomass microbiome studies (Fierer et al., 2025). Carryover prevention with uracil-N-glycosylase systems remains a best practice to limit amplicon contamination between runs (Mizumoto-Teramura et al., 2024). Assay design should rely on validated primer–probe sets with *in-silico* specificity checks and empirical verification (melt curves/amplicon sequencing); recent tools and updates facilitate rigorous, scalable *in-silico* screening (Collatz et al., 2025). Standard curves with explicit limits of detection/quantification, appropriate multiple-testing control, and conservative Ct thresholds are mandated by the updated MIQE 2.0 recommendations (Bustin et al., 2025). Finally, interpretation should be context-aware: where viability is uncertain, note that PMA/EMA-qPCR has important limitations and may only qualitatively distinguish live/dead under constrained conditions, warranting orthogonal confirmation (e.g., duplex targets or sequencing of representative positives) and, when possible, integration with metagenomic/spatial evidence and clinical phenotype (Kaur et al., 2024).

### 4.4 Fluorescence *in situ* hybridization

Fluorescence *in situ* hybridization (FISH) is a spatially resolved detection method that uses fluorescently labeled oligonucleotide probes to hybridize with microbial RNA or DNA within tissue samples. FISH allows direct visualization of microbial localization in CRC tissues, preserving spatial context and enabling co-staining with host markers (Yincharoen et al., 2025). Its integration with confocal microscopy or 3D imaging provides high-resolution insights into bacteria-host interactions, especially for low-biomass or intracellular organisms that may be underrepresented in sequencing data.

Recent adaptations, such as RNAscope-FISH, offer enhanced signal amplification and single-cell resolution, enabling the visualization of metabolically active tumor-associated bacteria (Castellarin et al., 2012; Strauss et al., 2011). However, this approach is largely limited to endpoint analyses and requires careful optimization of tissue processing and probe design.

### 4.5 Advanced imaging techniques

Modern imaging-based microbial profiling has further expanded the ability to study host–microbe interactions at high spatial resolution. Techniques such as immunohistochemistry (IHC), high-resolution electron microscopy (EM), and correlative focused ion beam/scanning electron microscopy (c-FIB/SEM) combine ultrastructural imaging with fluorescent microbial labeling, revealing 3D bacterial niches within tumor tissue (Weiner and Enninga, 2019).

Use of fluorochrome-conjugated, bacteria-specific antibodies and bacterial metabolic labeling, such as fluorescent D-alanine incorporation into bacterial cell walls, allows selective imaging of live, metabolically active bacteria in fresh tumor samples (Casasanta et al., 2020; Puschhof et al., 2021; Xue et al., 2023). These methods are particularly valuable for linking microbial spatial distribution to functional interactions, though they remain largely limited to research settings due to technical complexity and endpoint constraints.

### 4.6 Spatial transcriptomics and multi-omics profiling

Spatial transcriptomics and multi-omics integration now provide unprecedented resolution in mapping tumor-associated microbial niches. RNAscope-FISH allows single-cell bacterial RNA localization, while digital spatial profiling (GeoMX) simultaneously quantifies dozens of immune-related proteins, correlating microbial presence with local immune contexture.

The 10x Visium spatial transcriptomics platform enables host gene expression mapping *in situ*, linking microbial colonization to tumor margin characteristics, including hypovascular and immunosuppressive microenvironments (Galeano Niño et al., 2022). These integrated spatial approaches bridge microbial detection and functional tumor biology, revealing how localized microbial communities influence immune evasion, tumor progression, and therapy response.

## 5 Gut microbiota-based interventions in CRC therapy

With growing recognition of the gut microbiome as a pivotal modulator of host health, microbiota-targeted interventions have emerged as promising adjunct strategies in CRC management. Mounting evidence indicates that the gut microbial community not only contributes to tumor initiation and progression but also modulates responses to chemotherapy, immunotherapy, and other treatment modalities through its impact on drug metabolism, host immunity, and inflammatory signaling (Sánchez-Alcoholado et al., 2020; Kim and Lee, 2022; Kalasabail et al., 2021). Altered microbial profiles in CRC, characterized by enrichment of pro-carcinogenic species such as *F. nucleatum* and depletion of beneficial taxa like *Bifidobacterium*, have been associated with poor treatment responses and increased therapy-related toxicity (Fong et al., 2020). Conversely, specific beneficial microbes, including members of the *Bifidobacterium* genus, have been shown to enhance the efficacy of oxaliplatin and other chemotherapeutics, likely through immune modulation and reduction of intestinal inflammation (Chawrylak et al., 2024). In contrast, broad-spectrum antibiotic-induced dysbiosis can attenuate therapeutic efficacy and exacerbate adverse effects (Van Dingenen et al., 2023).

These observations underscore the concept that the “microbial status” of the gut is a determinant of therapeutic outcomes, providing a rationale for microbiota-targeted interventions. Currently, three major strategies have been investigated in preclinical and clinical settings: (i) probiotics and prebiotics, which support the growth and function of beneficial microbes; (ii) fecal microbiota transplantation (FMT), which reconstructs a balanced microbial ecosystem; and (iii) bacteriophage therapy, which selectively eliminates pro-carcinogenic or antibiotic-resistant bacteria (Masheghati et al., 2024; Zuo et al., 2024; Song et al., 2020).

### 5.1 Microbiota modulation of therapeutic responses

**Figure 2:**
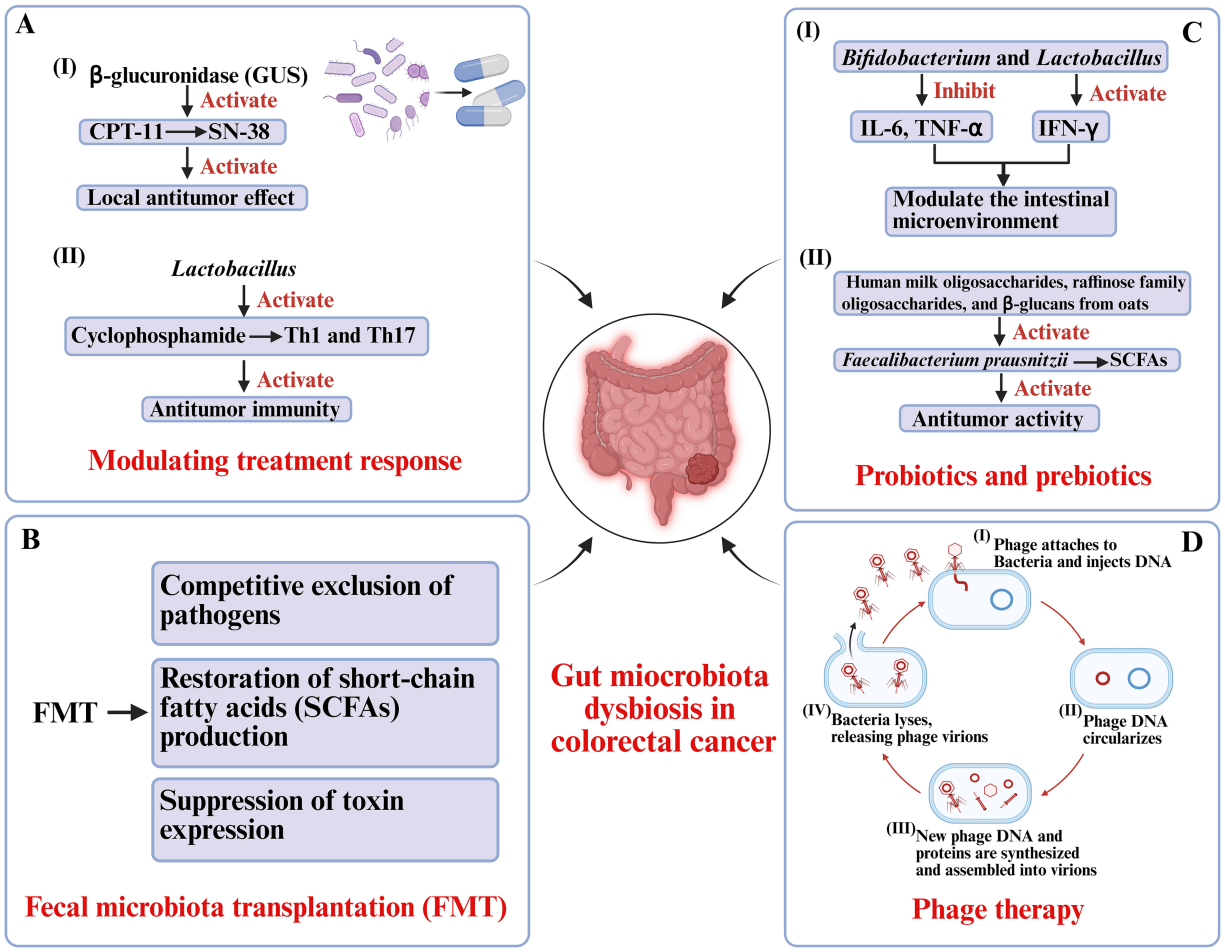
Microbiota-based therapeutic strategies in colorectal cancer. **(A)** Modulation of treatment responses: Gut microbial β-glucuronidase (GUS) reactivates irinotecan metabolite SN-38, enhancing local antitumor effects but contributing to toxicity. *Lactobacillus* augments cyclophosphamide-induced Th1/Th17 responses and antitumor immunity. **(B)** Fecal microbiota transplantation (FMT): Restores gut homeostasis by excluding pathogens, reestablishing short-chain fatty acid (SCFA) production, and suppressing toxin expression. **(C)** Probiotics and prebiotics: *Bifidobacterium* and *Lactobacillus* modulate cytokine expression, strengthen intestinal barrier function, and promote SCFA-producing bacteria (*Faecalibacterium prausnitzii*), supporting antitumor immunity. **(D)** Phage therapy: Bacteriophages selectively lyse pathogenic bacteria through infection cycles, reducing tumor-promoting microbes while sparing commensals.

The gut microbiome influences CRC therapy through multiple mechanisms, including regulation of drug metabolism, modulation of tumor immunity, and alteration of the inflammatory tumor microenvironment. For example, irinotecan (CPT-11), a widely used chemotherapeutic, requires conversion to its active metabolite SN-38, which is subsequently inactivated by glucuronidation in the liver. Gut microbial GUS can reactivate SN-38 in the colon, enhancing its local antitumor effect but simultaneously causing dose-limiting gastrointestinal toxicity (Bell et al., 2021; Yang Q. et al., 2023). Similarly, ROS generation induced by microbial modulation of NADPH oxidase activity is essential for the cytotoxic efficacy of platinum-based chemotherapies such as oxaliplatin (Figure 2A) (Cheng W. et al., 2020).

Microbiota-driven immune modulation also plays a crucial role. Certain commensals, such as *Lactobacillus* species, facilitate cyclophosphamide-induced Th1 and Th17 immune responses, enhancing antitumor immunity. *Barnesiella intestinihominis* has been shown to promote interferon-γ (IFN-γ) release and reduce regulatory T cell activity, creating a tumor-suppressive immune milieu (Aghamajidi and Vareki, 2022; Rad et al., 2024). Conversely, gut microbiota dysbiosis may attenuate therapeutic sensitivity and even contribute to treatment-related complications (Mohseni et al., 2023). These findings suggest that restoring or optimizing the gut microbial ecosystem could serve as a strategy to improve both the efficacy and safety of CRC therapy (Figure 2A).

### 5.2 Fecal microbiota transplantation

FMT represents a more comprehensive approach to restoring gut microbial homeostasis by transferring processed stool from healthy donors to recipients. This method allows for ecosystem-wide reconstruction of microbial networks, potentially reestablishing metabolic and immune functions disrupted in CRC (Andary et al., 2024). FMT has already achieved high cure rates in recurrent *Clostridioides difficile* infection, with mechanisms including competitive exclusion of pathogens, restoration of SCFA production, and suppression of toxin expression (Figure 2B) (Yadegar et al., 2024).

In oncology, preclinical studies have shown that antibiotic-induced microbiome disruption diminishes the efficacy of chemotherapeutic agents like oxaliplatin and cyclophosphamide, while FMT can reverse this effect and restore antitumor immunity (Yang Y. et al., 2024). Early clinical studies suggest that FMT may improve postoperative gut function, enhance epithelial barrier integrity, and modulate immune signaling pathways such as IL-10 and IFN-γ, potentially contributing to a less inflammatory tumor microenvironment (Xu et al., 2022).

For oncology applications, patient safety remains paramount. Key risks include pathogen transmission (e.g., multidrug-resistant organisms [MDROs]), bacteremia/sepsis, transmission of opportunistic or emerging agents, and horizontal gene transfer of antimicrobial-resistance genes. Additional concerns involve long-term engraftment stability, metabolic effects (e.g., unintended weight/metabolic shifts), and exacerbation of inflammation in vulnerable hosts (immunocompromised, mucosal injury) (Peery et al., 2024). Best practices therefore include (i) rigorous donor screening (travel/behavioral risks, comprehensive serology and stool pathogen panels with MDRO screening); (ii) standardized processing (closed systems, defined storage, traceability, batch QC/endotoxin testing); (iii) clear contraindications and informed consent; (iv) post-procedure pharmacovigilance with predefined adverse event (AE)/serious adverse event (SAE) reporting windows; and (v) registry-based long-term follow-up. For interventional trials, we recommend protocolized lot release criteria, recipient risk stratification, and co-primary safety endpoints (e.g., serious infection rate, MDRO colonization), alongside efficacy readouts.

### 5.3 Probiotics and prebiotics

Probiotics-live microorganisms that confer health benefits to the host-have demonstrated promising roles in CRC prevention and adjunct therapy. Species such as *Bifidobacterium* and *Lactobacillus* modulate the intestinal microenvironment by enhancing barrier integrity, downregulating pro-inflammatory cytokines (e.g., IL-6, TNF-α), and upregulating antitumor cytokines (e.g., IFN-γ) (Li et al., 2024). Preclinical studies using *Bifidobacterium longum* have shown delayed tumor growth, reduced inflammation, and restoration of epithelial barrier function in murine CRC models (Shang et al., 2024). Clinically, supplementation with *Lactobacillus rhamnosus* GG (LGG) for four weeks postoperatively reduced infection rates, decreased intestinal inflammation, and improved gut microbial diversity in CRC patients (Rafter et al., 2007). Mechanistically, these effects involve enhanced expression of tight junction proteins, promotion of epithelial repair, inhibition of pathogen adhesion, and stimulation of antimicrobial peptide secretion (Figure 2C) (Si et al., 2021).

Prebiotics-non-digestible substrates that selectively promote beneficial microbial growth-serve as “metabolic enhancers” for probiotics (Park et al., 2020). Compounds such as human milk oligosaccharides, raffinose family oligosaccharides, and β-glucans from oats have been shown to increase populations of SCFA-producing bacteria like *Faecalibacterium prausnitzii*. The SCFAs, particularly butyrate, maintain mucosal homeostasis, regulate immune responses, and suppress inflammation-driven tumorigenesis (Kok et al., 2020). Epidemiological data further support that diets rich in prebiotic fibers (e.g., whole grains, fruits, and vegetables) are associated with a reduced risk of CRC (Turati et al., 2022). Emerging “synbiotic” strategies that combine probiotics and prebiotics have demonstrated synergistic effects, enhancing microbial resilience and antitumor activity (Figure 2C) (Oh et al., 2020).

### 5.4 Bacteriophage therapy

Bacteriophages are viruses that specifically infect bacteria and can selectively target pro-carcinogenic or multidrug-resistant species in the gut (Danis-Wlodarczyk et al., 2021). Lytic phages bind bacterial surface receptors, replicate within the host, and induce bacterial lysis, thereby reducing pathogen burden and associated inflammatory signaling (Shahin et al., 2021). Compared with broad-spectrum antibiotics, phages offer precision targeting with minimal disruption to commensal microbes.

In CRC–relevant gut microbiota dysbiosis, phages can be engineered or selected to target defined taxa while preserving commensals. For example, phages directed against *F. nucleatum* or *E. coli* harboring the *pks* island (*pks*^+^ *E. coli*) may lower inflammatory signaling, curtail genotoxic potential, and mitigate therapy-interfering microbial functions. Such approaches can be integrated with chemotherapy or immunotherapy in biomarker-defined contexts to enhance therapeutic efficacy.

Successful translation must also account for phage–host coevolution and polymicrobial ecology. Bacteria can rapidly evolve receptor modifications, deploy restriction–modification or abortive-infection systems, and activate CRISPR–Cas defenses, which can shorten phage durability. Recent work underscores these arms-race dynamics and multi-defense synergies in natural and engineered settings (Chen et al., 2024). Countermeasures include rational phage cocktails that diversify receptor usage, adjuvants such as depolymerases and biofilm disruptors, and adaptive reformulation guided by longitudinal microbiome surveillance; contemporary reviews also highlight CRC-relevant opportunities and constraints for phage strategies (Mayorga-Ramos et al., 2024). In the polymicrobial, biofilm-rich microenvironments typical of CRC lesions, community interactions may impede phage adsorption or metabolically shield targets; phage-enabled and nanocomposite approaches are being explored to improve biofilm penetration (Mayorga-Ramos et al., 2024). Practical implications include (i) testing efficacy in community/biofilm models, (ii) optimizing dosing routes and pharmacokinetics for mucosal delivery (e.g., encapsulation, mucoadhesive matrices), (iii) monitoring neutralizing antibodies and mucin binding, and (iv) implementing rigorous quality control for manufacturing (potency, purity/endotoxin, stability); emerging clinical and pharmaceutical data emphasize antibody-mediated neutralization and formulation/stability requirements (Sawa et al., 2024). Thoughtful antibiotic–phage sequencing (to harness synergy and avoid antagonism) and real-time resistance surveillance should be embedded in trial designs to preserve activity and ecological balance; recent overviews of phage therapy for multidrug-resistant (MDR) infections and gastrointestinal (GI) contexts reinforce these design principles (Kim M. K. et al., 2025).

Although preclinical studies have shown encouraging antitumor potential, clinical translation remains constrained by host immune clearance, the emergence of bacterial resistance, and manufacturing standardization (Khalid et al., 2021). A deeper understanding of phage–microbiome–host interactions, together with integration of phage therapy into existing microbiota-based strategies, may open new therapeutic avenues for CRC (Figure 2D).

## 6 Translational landscape and challenges

Diagnostic tools: Multi-omics has matured from exploratory discovery to clinically actionable workflows. 16S rRNA sequencing and shotgun metagenomics are best positioned for initial screening and feature extraction, enabling strain-level detection of carcinogenic determinants (e.g., *pks*, *bft*) and cross-cohort reproducibility. Targeted qPCR panels provide rapid, low-cost surveillance for perioperative and longitudinal follow-up—well suited to monitoring *F. nucleatum*, *pks*^+^ *E. coli*, and enterotoxigenic *Bacteroides fragilis* in feces or tissue. For difficult or refractory cases, FISH and spatially resolved profiling (e.g., RNAscope, digital spatial proteomics) localize microbes within tumor–immune niches and link presence to pathway activity. Practically, we recommend high-throughput sequencing for discovery and risk stratification, qPCR for routine monitoring, and tissue imaging when spatial context will change management (Table 1).

Interventional measures: Probiotics and prebiotics can reinforce barrier integrity, temper mucosal inflammation, and restore SCFA production; they are suitable as adjuncts around surgery or during chemotherapy to reduce gastrointestinal toxicity. FMT shows signals for postoperative functional recovery and immune modulation, but translation hinges on stringent donor screening, standardized processing, and pharmacovigilance for long-term safety. Bacteriophage (phage) therapy offers precision removal of pro-carcinogenic taxa (e.g., *F. nucleatum* or *pks*^+^ *E. coli*) with minimal off-target disruption, yet faces hurdles including host immune clearance, resistance, and scalable manufacturing. A pragmatic clinical algorithm is to prioritize (i) anti-inflammatory and antimicrobial adjuncts when *F. nucleatum* burden is high; (ii) β-glucuronidase (GUS) inhibition and toxicity management when *pks*/colibactin signatures are present; and (iii) consider phage or targeted antimicrobial strategies in biomarker-defined, refractory microbiome states.

Challenges and proposed solutions: (1) Causality and trial design—move beyond association by embedding microbial mechanisms into eligibility and randomization: enroll by “microbe–pathway–therapy” matches (e.g., *F. nucleatum*-high → antibiotic/phage + chemotherapy; *pks*^+^ → irinotecan-GUS mitigation bundles) and power trials for DFS/OS as well as toxicity endpoints. (2) Standardization—harmonize biospecimen handling, sequencing/qPCR pipelines, and reporting standards to enable multi-center validation and regulatory review. (3) Safety and durability—establish long-term surveillance for microbiome interventions (FMT, phage, high-dose probiotics), including resistance and horizontal gene transfer monitoring. (4) Minimal, deployable multi-omics panels—co-develop small, cost-effective signatures (metagenomics + metabolomics + qPCR) that track with clinical endpoints (DFS, OS, adverse events) and integrate into perioperative and adjuvant treatment pathways. Together, these steps outline a feasible road map toward microbiome-informed precision oncology.

## 7 Conclusions and future perspectives

The intricate relationship between the gut microbiota and CRC has gained considerable attention in recent years, revealing profound implications for tumor biology, diagnosis, and therapy. Accumulating evidence indicates that the gut microbiome contributes to CRC initiation, progression, and prognosis through multiple interrelated mechanisms, including chronic inflammation, bacterial genotoxin production, oxidative stress, and aberrant microbial metabolism (Png et al., 2022). Specific taxa, such as *F. nucleatum*, *E. coli*, and *B. fragilis*, are frequently enriched within tumor tissues, and their high abundance has been correlated with poor clinical outcomes, highlighting their potential roles as biomarkers and therapeutic targets (Alexander et al., 2023). At the same time, heterogeneity across studies—driven by differences in sampling, sequencing, and analysis pipelines, as well as confounding influences of diet, antibiotic exposure, and host genetics—complicates the interpretation of associations and underscores the need for methodological rigor and standardization (Diacova et al., 2025).

Advances in high-throughput sequencing, multi-omics integration, and bioinformatics have greatly enhanced our ability to characterize gut microbial composition and function with increasing precision, enabling earlier detection of dysbiosis, improved prediction of therapeutic responses, and movement toward individualized interventions in CRC. Recent multi-omics studies and reviews illustrate prognostic modeling and subtype discovery, as well as clinical screening potential, when metagenomics is integrated with metabolomics/proteomics and other layers (Xu et al., 2024). However, variability in pre-analytical procedures (e.g., stool collection/stabilization/storage) and platform effects (targeted amplicon vs. shotgun metagenomics) can introduce batch effects and limit cross-cohort comparability. Comparative work from 2024 to 2025 underscores method-dependent differences between 16S and shotgun approaches, while studies on FIT-derived material, domestic freezer storage, and field-collection protocols highlight how handling choices shape profiles (Bars-Cortina et al., 2024). Downstream bioinformatics also contributes to between-study variability, motivating updated computational best practices (Pita-Galeana et al., 2025). Addressing these issues will require harmonized protocols, shared reference materials, and transparent reporting standards (e.g., STORMS and related community efforts), alongside systematic capture of key covariates—particularly detailed diet and medication histories and host genomic data—to enable robust adjustment for confounding; recent large-scale analyses further emphasize microbial load, diet, and medications as major drivers of variability (Forry et al., 2024).

Microbiota-targeted interventions—including probiotics, prebiotics, FMT, and bacteriophage therapy—are emerging as promising strategies for both CRC prevention and adjunctive treatment (Lei et al., 2025). These approaches aim to restore microbial homeostasis, reshape the tumor microenvironment, and enhance the efficacy of existing therapeutic modalities, providing new opportunities for precision oncology. Nonetheless, considerable interindividual variability in treatment response is consistently observed. Sources of this variability likely include baseline community structure and function, host immune tone, mucosal ecology (e.g., biofilms), diet, recent antibiotic exposure, and host genetic variation in pathways mediating microbe–host interactions (Kim K. et al., 2025). Prospective trials should therefore incorporate responder/non-responder stratification, dietary control or standardized counseling, pre-specified antibiotic washout periods where feasible, and integration of host multi-omics to identify predictive biomarkers and guide patient selection.

To make these advances clinically actionable, we outline a multi-omics–to-clinic framework. We prioritize a minimal diagnostic signature that pairs metagenomics (with optional metabolomics) and targeted qPCR to deliver reproducible, cost-aware risk stratification. We then implement a pathway–therapy–microbiome matching schema that aligns dominant microbial mechanisms (genotoxin production, chronic inflammation, metabolite dysregulation, biofilm ecology) with tailored interventions (e.g., β-glucuronidase mitigation, anti-inflammatory adjuncts, probiotics/prebiotics, FMT, or phage targeting of *F. nucleatum* and *pks^+^ E. coli*). Finally, we recommend a three-step clinical path—stratified diagnosis → therapy matching → longitudinal monitoring—to support prospective validation, safety/quality oversight, and real-time adaptation based on microbiome dynamics.

Several critical knowledge gaps remain. First, establishing causal relationships between gut microbes and CRC is essential, necessitating well-designed *in vivo* and *ex vivo* models to move beyond correlative studies (Liu et al., 2023). Complementary causal-inference approaches (e.g., longitudinal designs, mediation analyses) may help disentangle confounding by diet, antibiotics, and host genetics. Second, there is an urgent need for highly sensitive, specific, and cost-effective diagnostic tools to facilitate the clinical implementation of microbiome-based biomarkers (Kværner et al., 2021). Such tools should be validated across centers using standardized workflows, external quality controls, and comprehensive metadata capture to ensure generalizability. Third, the safety, efficacy, and durability of microbiota-targeted interventions require validation in large, multicenter clinical trials, with careful attention to interindividual variability, host–microbiome interactions, and the potential ecological trade-offs of therapy (Huang et al., 2022). Pragmatic trial designs, real-time resistance/ecology surveillance (for antibiotics and phages), and consensus manufacturing/quality criteria (potency, purity, stability, endotoxin burden) will be important for translation.

Looking forward, the integration of advanced technologies such as artificial intelligence (AI) and machine learning with multi-omics datasets—encompassing microbiome, metabolome, genome, and single-cell transcriptome—offers an unprecedented opportunity to build predictive models for CRC risk, prognosis, and therapeutic response (Fusco et al., 2024; Yang L. et al., 2023). To realize this potential, models must be trained on well-annotated, harmonized datasets that include standardized laboratory and computational pipelines and rich covariate metadata (diet, medications, host genetics). AI-driven feature extraction can accelerate the identification of microbial signatures for precision diagnostics, while deep learning approaches may elucidate complex host–microbiota network interactions, informing personalized treatment strategies (Zhang J. et al., 2024; Zhou et al., 2022). Equally important are prospective external validation, assessment of model transportability across populations and diets, and interpretable frameworks that link features to mechanism and actionability.

Overall, gut microbiome research is transitioning from descriptive studies toward precision applications in oncology. Realizing the promise of “microbiome-informed precision oncology” will depend not only on mechanistic insight and therapeutic innovation but also on field-wide standardization, rigorous control of confounding, and deliberate accommodation of interindividual variability in trial design and clinical implementation. By coupling methodological best practices with mechanistic and translational advances, microbiota-based diagnostics and therapeutics are poised to become integral components of comprehensive CRC management.
